# New distribution record, morphological and molecular characterization of *Dirofilaria (Nochtiella) tenuis* (Nematoda: Onchocercidae) in raccoons (*Procyon lotor)* from the Yucatan Peninsula, Mexico

**DOI:** 10.1016/j.ijppaw.2024.100981

**Published:** 2024-08-29

**Authors:** Aarón Hernández-Núñez, Víctor M. Vidal-Martínez, M. Leopoldina Aguirre-Macedo

**Affiliations:** Centro de Investigación y de Estudios Avanzados del Instituto Politécnico Nacional, Unidad Mérida, Departamento Recursos del Mar, Carretera Antigua a Progreso Km 6, Cordemex, Mérida, Yucatán, C.P. 97310, Mexico

**Keywords:** *Nematoda*, *Dirofilaria*, *Tenuis*, *Raccoon*, *Yucatan*, Mexico

## Abstract

*Dirofilaria (Nochtiella) tenuis* is a mosquito-borne subcutaneous parasite of raccoons, regarded as the causative agent of most human dirofilarial infections in North America. Despite the wide geographic range of raccoons in the Americas, the presence of this parasite has not been confirmed outside its known endemic areas in the Southern United States. Based on morphological and molecular data, we present the first record of *D. (N.) tenuis* in wild raccoons from the Yucatan Peninsula. Adult thread-like worms recovered from subcutaneous tissues of wild raccoons were analyzed with light microscopy, histology, scanning electron microscopy (SEM), and 18S rRNA, 28S rRNA and cox1 gene sequencing for identification and phylogenetic analysis. The collected nematodes were identified as *D. (N*.*) tenuis based on their morphology*. SEM analysis revealed details about different facial ornamentations in male worms, which had not been previously described. Molecular and phylogenetic analyses confirmed morphological observations by placing our specimens within clades of the *Dirofilaria* genus. Our findings represent the first molecular characterization for this nematode and extend the geographical range of this parasite to Mexico. Further studies are required for a more accurate picture of the epidemiology of this filarioid across Mexico and other areas overlapping the raccoon's range.

## Highlights

• First record of *Dirofilaria. (Nochtiella.) tenuis* infecting a mammal from Mexico.
• The morphology of male and female adult worms is presented.
• The first molecular characterization *Dirofilaria. (Nochtiella.) tenuis* is provided.
• The geographical range of *Dirofilaria. (Nochtiella.) tenuis* is extended to Mexico.

## Graphical abstract

**Figure:**
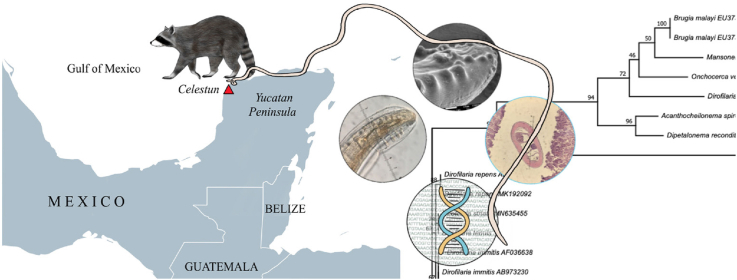

## 1 Introduction

Zoonotic parasitic diseases pose significant threats to wild and domestic mammals, as well as human health worldwide (Simón et al., 2017). The generic name Dirofilariasis combines a group of vector-borne diseases caused by species belonging to the genus *Dirofilaria* Railliet and Henry, 1911 (*Spirurida: Onchocercidae*) in wild carnivores, domestic dogs and cats, and humans (Simón et al., 2012; Harizanov et al., 2014). The genus comprises at least 27 valid species, divided into two subgenera, *Dirofilaria* and *Nochtiella* (Dantas-Torres and Otranto, 2013). *Dirofilaria* (*Dirofilaria*) encloses five species with an affinity for the cardiovascular system, including the cosmopolitan canine heartworm *Dirofilaria immitis* (Diaz, 2015). On the other hand, the subgenus *Dirofilaria* (*Nochtiella)* Faust, 1937 consists of 22 species that parasitize subcutaneous and conjunctival tissues (Gustinelli et al., 2012). Among these, *Dirofilaria (Nochtiella) tenuis* Chandler (1942), a parasite of the North American raccoon (*Procyon lotor*). Historically, *D. (N.) tenuis* has been considered endemic in areas of the Gulf Coast, from Texas to Florida, where it has been identified as a prevalent parasite in raccoon populations (Houston and Parks, 2022). This nematode is also regarded as one of the primary agents of human filarial infections in the United States (Vincent et al., 2013), making it the third most common *Dirofilaria* species reported in humans (Perles et al., 2024).

The life cycle of *D. (N.) tenuis* follows the typical pattern of dirofilarial nematodes, involving culicid mosquitoes (intermediate hosts and vectors) and the raccoon acting as the definitive host (Gustinelli et al., 2012). Typically, dioecious adult worms reside between the raccoon's adipose and subdermal reticular tissues (Collins et al., 1993). Viviparous females produce microfilariae, which migrate to the host's bloodstream, making them readily accessible to mosquitoes. Within the Malpighian tubules of the mosquitoes, the microfilariae develop into infective third-stage larvae (L3) (Pistey, 1958). During a blood meal, mosquitoes deposit hemolymph with L3 larvae, which penetrate the host's skin via the mosquito bite (Theis, 2005) and migrate through subcutaneous tissues to mature and reproduce.

Raccoons are native mesocarnivores of Central and North America, with the most widespread distribution among the Procyonidae, ranging from Panama to Canada and Alaska (Gehrt, 2003). Although helminth parasites of the raccoon are relatively well documented in North America (Richardson, 2013; Weinstein et al., 2019), data on the occurrence and prevalence of raccoon parasites outside the United States and Canada remain limited. Herein, we document, for the first time, the presence of *Dirofilaria (N.) tenuis* in raccoons from Yucatan, Mexico. We also provide new morphological data from light microscopy and SEM image analysis, along with the first amplification and phylogenetic analysis of the 18S rRNA, 28S rRNA and cox1 genes for the species.

## 2 Materials and methods

This study was conducted between 2022 and 2023 in the Ria Celestún Biosphere Reserve (RCBR) (20° 59′ 33.72″ N and 90° 31′ 13.15″ W), located in the northwestern portion of the Yucatan Peninsula, Mexico. Five adult raccoons were collected under the scientific collector license (SPARN/DGVS/02225/22) issued by the Ministry of Environment and Natural Resources of Mexico (SEMARNAT) under the Official Mexican Standard (NOM-033-SAG/ZOO-2014) for wild and domestic animals sacrifice and the AVMA Guidelines for the Euthanasia of Animals (Sikes, 2016). Raccoons were dissected, and the subcutaneous tissues, skeletal muscle, body cavities, and organs were inspected for parasites using a stereo microscope (Motic SMZ-168, Japan). The collected nematodes were washed in saline (0.9% NaCl) and fixed in 4% formalin, AFA, or 70% ethanol for morphological, histological and SEM studies. For DNA isolation, we used 96% ethanol.

### 2.1 Morphology under light microscopy

Nematode specimens were cleared using a series of 70% ethanol and glycerol concentrations and then examined under light microscopy (Leica DM2500, Germany). Measurements were made directly with an eyepiece micrometer or estimated with the image software LAS V4.5 (Leica Microsystems, https://www.leica-microsystems.com/). The range of each measurement is provided, followed by the mean and standard deviation (mean ± SD) in micrometers unless otherwise indicated. Photographs of mounted nematodes were obtained using a Leica MC170 HD digital camera. Parasites were identified using available morphological descriptions by Chandler (1942), Orihel and Beaver (1965), and Gutierrez (1984).

### 2.2 Histology

Standard histological procedures were employed to prepare paraffin blocks using a graded series of ethanol, chloroform, and paraffin to verify the presence of Longitudinal Cuticular Ridges (LCR) and assess their shape, length, and width, as well as other morphological diagnostic features, allowing discrimination between the subgenera and species of *Dirofilaria*. Midbody sections (10 mm) of male and female specimens, fixed in Davidsons AFA (acetic acid, formaldehyde, alcohol), were positioned upright between slices of chicken liver (preserved in 10% buffered formaldehyde). Paraffin blocks were then cross-sectioned, obtaining 5 μm thick slices with a microtome (KD-3358, Zhejiang Jinhua Kedi Instrumental Equipment, China). The resulting sections were mounted on glass slides, stained with hematoxylin and eosin (HE), embedded in Canada balsam, and examined under a light microscope (Leica DM2500, Germany). Photographs were obtained with a Leica MC170 HD digital camera connected to the microscope and analyzed with the image software LAS V4.5 (Leica Microsystems, https://www.leica-microsystems.com/).

### 2.3 Scanning electron microscopy (SEM)

Specimens intended for SEM were gradually dehydrated in an ethanol concentrations series (70–100%). Subsequently, 2 cm long sections from the anterior and posterior ends of three female and three male worms were critical-point dried in liquid CO_2_ using a K850 Critical Point Dryer (Quorum Technologies, UK). The dried sections were stored at 24 °C in a silica gel desiccator. For SEM analysis, specimens were mounted onto stubs using conductive double-sided adhesive tape and then sputter-coated for 40 s with gold/palladium (Au/Pd) in a Q150R Plus Metallizer (Quorum Technologies, UK) and examined in a Jeol-7600F scanning electron microscope (Jeol, Tokyo, Japan) at Cinvestav-Merida.

### 2.4 Molecular-based analysis

Genomic DNA was extracted from adult female nematodes following the provider's animal tissues protocol (DNeasy® Blood & Tissue (QIAGEN, Hilden, Germany) handbook (HB-2061-003). PCR amplification was performed using tree primer sets: Diro18S-F1 (5′-CCATGCATGTCTAAGTTCAA–3′)/R1(5′-TCGCTACGGTCCAAGAATTT–3′) and Diro18S-F2 (5′-CTGAATACTCGTGCATGGAA–3′)/R2(5′-TTACGACTTTTGCCCGGTT–3′) for 18S rRNA gene (Suzuki et al., 2015), 391 (5′ AGCGGAGGAAAAGAAACTAA–3′ (Nadler and Hudspeth, 1998) 536 5′–CAGCTATCCTGAGGGAAAC–3′ (García-Varela and Nadler, 2005) for the 28S rRNA gene, and for cox1 COIintF (5′-TGATTGGTGGTTTTGGTAA-3′) and COIintR (5′-TAAGTACGAGTATCAATATC-3′) (Casiraghi et al., 2001). All PCR reactions were carried out in a final volume of 25 μl, containing 12.5 μl Green GoTaq Master Mix (Promega, Madison, WI, USA), 1 μl of each primer (10 μM), 8.5 μl distilled water and 2 μl of gDNA for the 18s and 28s rRNA. For the cox1, the PCR mix contained 0.5uL of each primer (10 μM). The 18S rRNA and cox1 reactions were performed in a Veriti-AB thermocycler (Applied Biosystems Veriti ABI Inc., CA, USA) The program for 18S rRNA consisted in an initial denaturation at 94 °C for 5 min, followed by 30 cycles of amplification, denaturation at 94 °C for 30 s, annealing at 60 °C for 30 s, extension at 72 °C for 1 min, and a final extension at 72 °C for 5 min. The program for cox1 gene fragment amplification consisted of denaturation at 94 °C for 5 min, followed by 40 cycles of denaturation at 94 °C for 45s, 52 °C for 45s, 72 °C for 90s, and a final extension at 72 °C for 5 min. The 28S rRNA PCR reactions were performed in an Axygen® MaxyGene™ II thermocycler1 (Corning, New York, USA) under the following conditions: initial denaturation at 94 °C for 5 min, followed by 35 cycles of amplification, denaturation at 94 °C for 1 min, annealing at 50 °C for 1 min, extension at 72 °C for 1 min, and finally an extension at 72 °C for 10 min.

All the PCR products were analyzed by 1% agarose gel electrophoresis with 1X TAE buffer, stained with RedGel (Biotium, San Francisco, USA), and observed under ultraviolet light with the BioDoc-It2 Imager photodocumenter (Analytik Jena AG, Thuringia, Germany). The 18S rRNA and cox1 PCR products were carried out by Sanger sequencing at Azenta Life Sciences (South Plainfield, NJ, USA; https://www.azenta.com/). To confirm the middle of the sequence of the 28S gene, we used the internal primers 503 (5′–CCTTGGTCCGTGTTTCAAGACG–3′) (Stock et al., 2001) and 504 (5′–CGTCTTGAAACACGGACTAAGG–3′) (García-Varela and Nadler, 2005). Purification and sequencing of the 28S rRNA PCR products were performed at the Instituto de Biología, UNAM, Mexico. The resulting sequences were analyzed and edited using Geneious Pro 4.8.4 software (Biomatters Ltd., Auckland, New Zealand). The obtained consensus sequences were subjected to preliminary analysis using the Basic Local Alignment Search Tool (https://blast.ncbi.nlm.nih.gov/Blast.cgi).

### 2.5 Phylogenetic analysis

In order to determine species identity, obtained 18S, 28S rRNA and cox1 sequences were compared with nucleotide records in the GenBank database using the online NCBI BLASTN suite (https://blast.ncbi.nlm.nih.gov/Blast.cgi). Sequences were aligned with the Multiple Sequence Comparison by Log-Expectation (MUSCLE) v3.8 program (Edgar, 2004) using the following command line: *muscle -infile -outfile -maxiters 16 -diags* in the MacOS Terminal v2.11 (Apple Inc, 2023). Aligned sequences were then trimmed at their ends to remove unaligned or ambiguously aligned regions using Geneious v.11.0.18. Sequences from this study were deposited into GenBank (accession nos: PQ248142, PQ248143 and PQ219693).

Phylogenetic analyses were conducted for each *D.* (*N*.) sequence. The 18S rRNA tree was inferred using the Maximum Likelihood method and Tamura 3-parameter model (Tamura et al., 2021) with a discrete Gamma distribution to model evolutionary rate differences among sites. The following sequences were used: *Achanthocheilonema spirocauda* (HG005138), *Brugia malayi* (EU373610, EU373618), *Dipetalonema reconditum* (AF217801) *D. immitis* (OP81190, AF036638, AB973230 and AB973231), *Dirofilaria repens* (AB973229 and MK192092), *Dirofilaria striata* (MN635455), *Dirofilaria ursi* (LC570022), *Litomosoides sigmodontonis* (AF227233), *Mansonella ozzardi* (AF228564), *Onchoceca volvulus* (EU272179), and *Dracunculus insignis* (AY947719) as outgroup. The phylogenetic analysis of 28S rRNA sequences was conducted using the Minimum Evolution (ME) method (Rzhetsky and Nei, 1992) with a pairwise deletion option (Felsenstein, 1985). The analysis was performed using the Close-Neighbor-Interchange (CNI) algorithm (Nei and Kumar, 2000) at a search level of 1. Sequences from *Brugia timory* (KP760362), *Brugia malayi* (KP760362), *Dipetalonema dracunculoides* (KP420152), *Dipetalonema reconditum* (AF217801), *Dirofilaria immitis* (KY990015), *Dirofilaria repens* (KP760376), *Loa loa* (KP760386), *Manzonella ozzardi* (KP760390), *Onchocerca volvulus* (AF228576), *Onchocerca gibsoni* (DQ317639), and *Wuchereria bancrofti* (EU370161) were used, with *Dracunculus* sp. (KY990016) as the outgroup.

For the cox1 sequences a maximum likelihood tree was constructed using the following sequences: *Acanthocheilonema spirocauda* (HF583266), *Brugia malayi* (MK250713), *Cercopithifilaria bainae* (MH390716), *Dipetalonema gracile* (AJ544877), Dirofilaria immitis (KF692100, OR434081), *Dirofilaria lutrae* (MK032318), *Dirofilaria repens* (MN200335, AJ271614), *Dirofilaria striata* (MN635457), *Dracunculus insignis* (EU646534), *Mansonella ozzardi* (JF412347), *Onchocerca volvulus* (MH190075), and *Dirofilaria ursi* (KY828980), with *Ascaris lumbricoides* (AB591796) as the outgroup. Phylogenetic trees were constructed in MEGAX. The best evolutionary models were chosen under the Bayesian Information Criterion (BIC) using the Mega software, and the statistical support was evaluated using 1000 bootstrap iterations.

## 3 Results

### 3.1 Morphological analysis

**Fig. 1:**
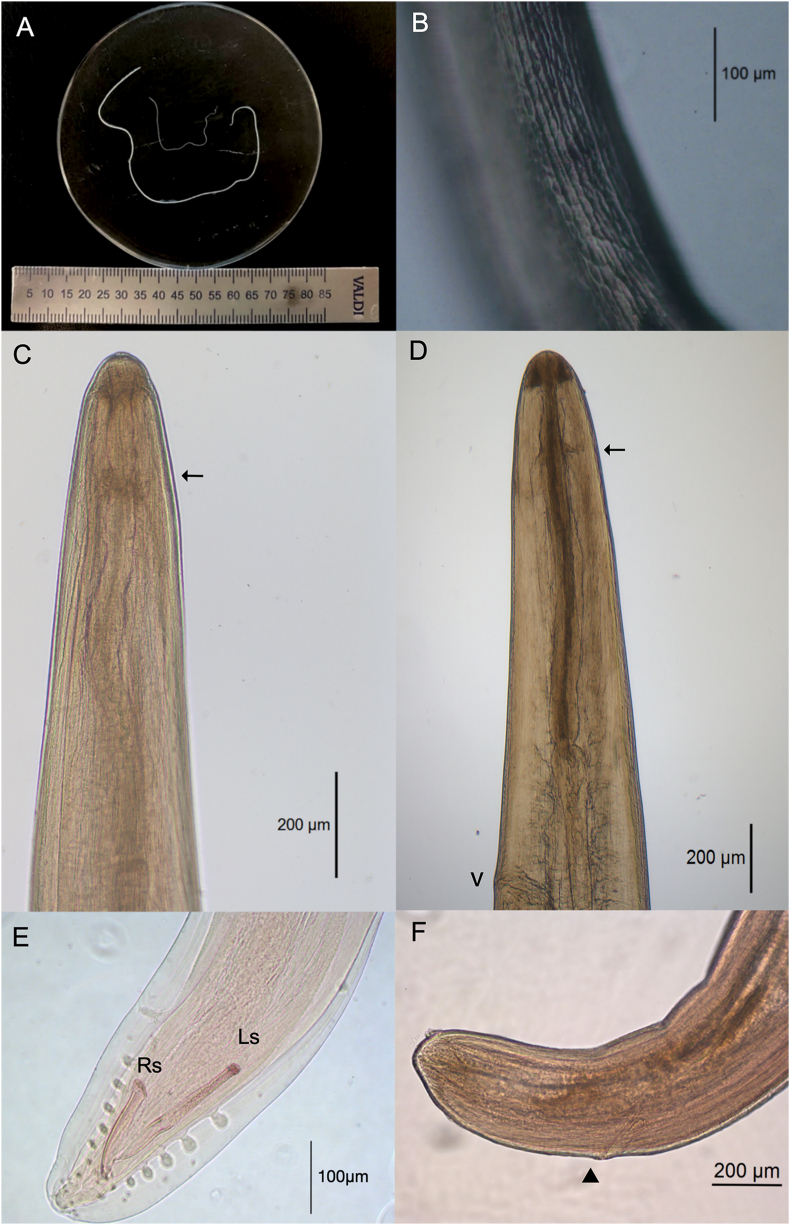
***Dirofilaria (Nochtiella) tenuis* from *Procyon lotor*.** Adult male and female worms (A). LCR under an optical microscope (B). Male, anterior portion of the body showing nerve ring (black arrow) (C). Female, anterior portion of the body showing nerve ring (black arrow) and vulva (v) (D). Male posterior end showing caudal papillae, right (Rs) and left (Ls) spicules (E). Female posterior end of the body showing the anal orifice (arrowhead) (F).

Body cylindrical, elongated with a whitish appearance, fairly uniform in diameter with cephalic and caudal ends tapered and rounded, tail short and mildly bent ventrally (Fig. 1C–F). Female worms 2.6 times larger than males (Fig. 1A). Both sexes had the typical cuticular ornamentations of the subgenus *Nochtiella* Faust, 1937, with fine cuticular transversal striations and longitudinal cuticular ridges in broken and branched patterns, readily observable in nonpermanent slides without coverslip and mounting media (Fig. 1B). Microfilariae (n = 5), extracted from the uteri near the vagina of a specimen preserved in 70% ethanol, unsheathed, finely striated transversely, with a roundish cephalic end and filamentous tail. Mounted in glycerol ranged 352 to 370 in length and 6.4 in width.

Males (n = 13): Body 30 mm–42 mm (38.8 ± 3.38) long, 200 to 270 (229 ± 23) wide at midbody. Nerve ring 220 to 280 (244.2 ± 14.1) from the anterior end (Fig. 1C). Esophagus 850 to 1025 (941 ± 65.6) long. Tail 70 to 80 (76.9) long. Posterior end conical coiled, with three to four progressively smaller turns. Caudal alae supported by a variable and unequal number of pedunculated preanal papillae, which ranged from 4 to 7 on both sides, decreased in size posteriorly, with a nearly symmetrical arrangement towards the anus; a pair of adanal and three to four postanal papillae, in addition to one large, lenticular-shaped, preanal papilla and a pair of small sessile papillae in the postanal midline (Fig. 1E). Phasmids near the tail tip, in anteroventral position. Copulatory spicules slightly curved ventrally, unequal, and dissimilar. Left spicule 200 to 225 (212 ± 7.62) long, divided into a slender tubular calamus, proximal portion, and a lamina ending in needle-shaped tip as distal segment. Right spicule 107 to 132 (121 ± 7.81), naviculate, short and wide with a rounded tip (Fig. 1E), anus 70 to 80 (76.9) from the posterior end, gubernaculum absent.

Females (n = 9): 78 mm–120 mm (104.5 ± 12.52) long, 320 to 420 (373 ± 32.81) wide at mid-body. Nerve ring 230 to 310 (262.5 ± 24.92) from the anterior end. Esophagus 920 to 1280 (1080 ± 118) long. Vulva, posterior to esophagus/intestine junction, 1125 to 3150 (1658 ± 672) from the anterior end. Vagina directed posteriorly (Fig. 1D), with two parallel ovaries, directed posteriorly, anus 195 to 240 (215.6 ± 15.90) from the posterior end (Fig. 1F).

### 3.2 Scanning electron microscopy

**Fig. 2:**
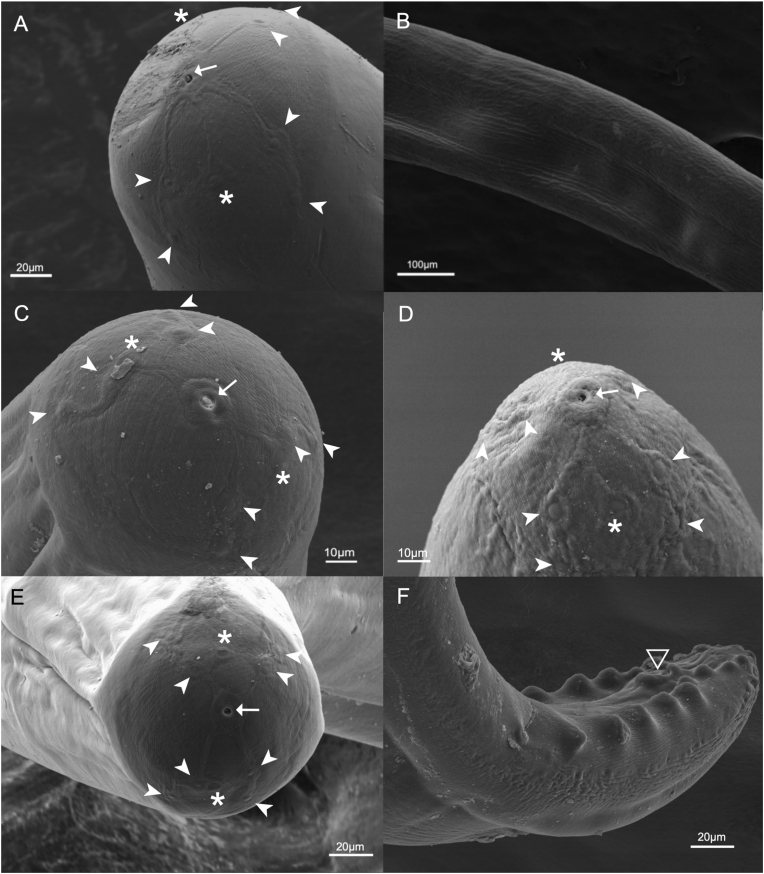
**Scanning electron microscopy of adult *Dirofilaria* (*Nochtiella*) *tenuis***. Anterior region of female (A) and male (C, D, and E), showing oral opening (arrow), 4 pairs of cephalic papillae (arrowheads), and a pair of amphids (asterisk). Longitudinal Cuticular Ridges (LCR) (B). Male posterior end (F) showing caudal papillae and cloaca (triangle).

Scanning Electron Microscopy analyses of the cephalic extremity showed a pattern of random cuticular striations in male and female specimens, approximately 1 μm wide, resembling fingerprints. The terminal oral opening appeared circular and lacked lips, 4 pairs of small cephalic papillae, and 2 lateral amphids (Fig. 2). The female head displayed an arrangement of ridges resembling a 6-pointed star-like shape, radiating from a raised rim surrounding the mouth region towards the cephalic papillae and amphidial pores (Fig. 2A). Males and females displayed, across most of their bodies, a series of wavy, broken, and branched longitudinal cuticular ridges (LCR), 4–6 μm wide, separated by 3–5 μm (Fig. 2B). These LCRs were composed of small units interrupted by transverse striations, resembling strings of square beads, which were less noticeable towards the terminal ends. Male specimens displayed three different facial ridges arrangements; in the first one, the rim surrounding the mouth gave rise to two lateral ridges that bifurcate and extend towards the papillae and the amphidial pores (Fig. 2C). In the second and third arrangements, the rim gave rise to 4 ridges that extend toward the papillae and the amphidial pores (Fig. 2D–E). Additional illustrations of some diagnostic structures from our specimens, as well as the observed ornamentation on the faces of females and males, are presented in supplementary material (S1).

### 3.3 Histology

**Fig. 3:**
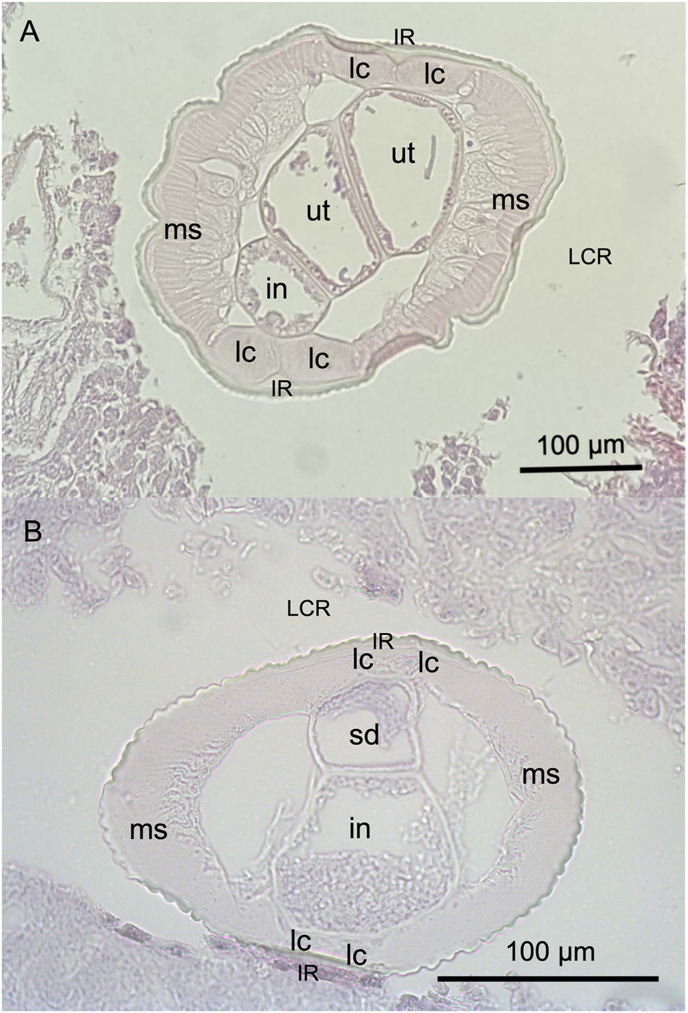
**Hematoxylin and eosin stain transverse sections of adult female (A) and male (B) worms at midbody**: in, intestine; IR, internal ridge, lc, lateral chords; LCR, Longitudinal Cuticular Ridges; ms, muscle; ut, uterus; sd; seminal duct.

Histologic transverse sections showed that the nematodes had a tick multilayered cuticle (6–8 μm females, 4–6 μm males). The cuticular layer in females (Fig. 3A) exhibited approximately 90 smoothly rounded longitudinal ridges (indentations), and 80 in males (Fig. 3B). Below the cuticle, a thick muscle layer —comprised of polymyarian muscle fibres— was divided by the lateral chords into the dorsal and ventral muscles.

### 3.4 Molecular analyses

**Fig. 4:**
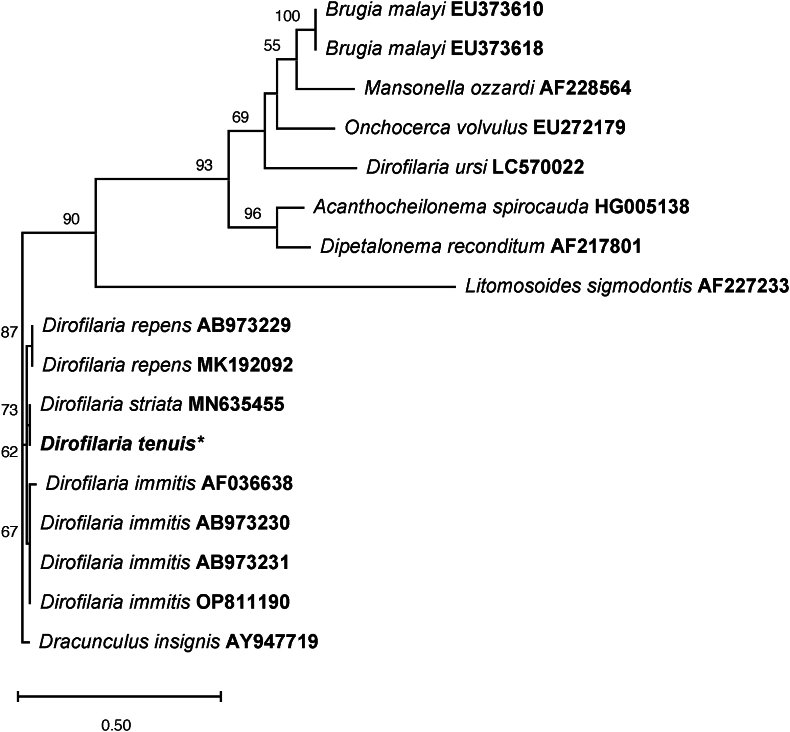
**Maximum likelihood tree based on partial 18S sequences of *Dirofilaria* spp. and some Onchocercidae nematodes**. Confidence support for the branches (1000 replicates) is shown as a percentage next to nodes. The units for the scale bar represent substitutions per site. The Dirofilaria tenuis sequence (*) of the specimen sequenced in this study. Labels after species names represent GenBank accession numbers of the sequences used for phylogenetic analysis.

**Fig. 5:**
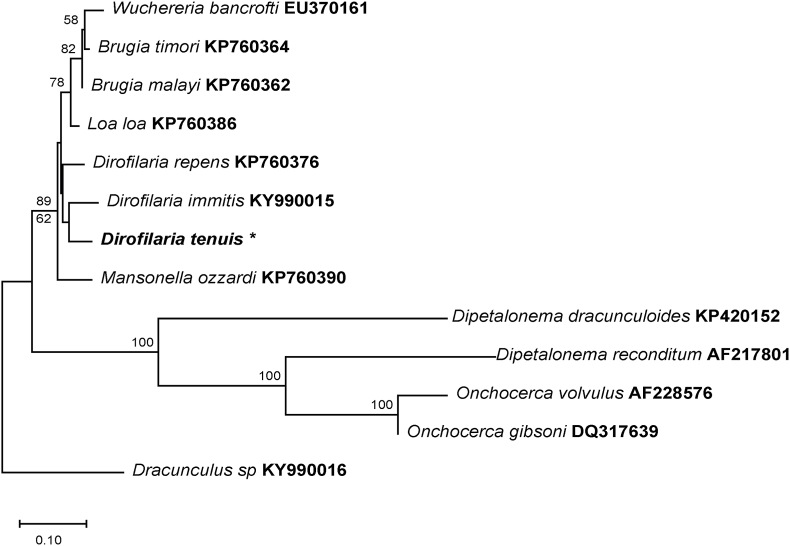
**Minimum Evolution tree based on partial 28S sequences of *Dirofilaria* spp. and some *Onchocercidae* nematodes**. Confidence support for the branches (1000 replicates) is shown as a percentage next to nodes. The units for the scale bar represent substitutions per site. Sequence with an asterisk (*) was derived from the Dirofilarial worms from raccoons in the current study. Labels after species names represent GenBank accession numbers of the sequences used for phylogenetic analysis.

**Fig. 6:**
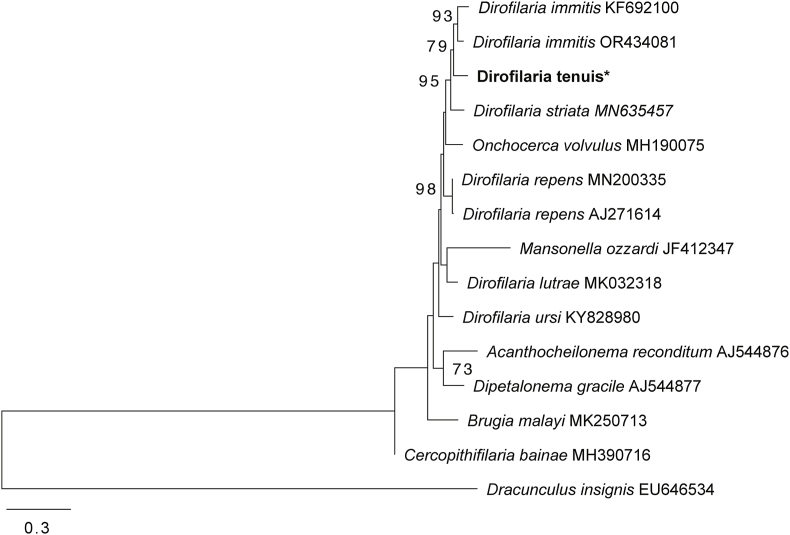
**Maximum likelihood tree based on partial cox1 sequences of *Dirofilaria* spp. and some Onchocercidae nematodes.** Labels after species names represent sequence GenBank accession numbers. Confidence support for the branches (1000 replicates) is shown as a percentage next to nodes. The units for the scale bar represent substitutions per site. The cox1 sequence of the present study is highlighted in bold and marked with an asterisk (*).

Pairwise identities, as determined by BLASTN, showed that the present 18S rRNA gene sequence (1603bp, GenBank accession no: PQ248143) shared 95.86% identity with *D. striata* (MN635455) with a query coverage (QC) of 50%, isolated from a domestic cat in Florida, USA. On the other hand, the 28S gene sequence (1210bp, GenBank accession no: PQ248142) exhibited 93.99% identity with *D. immitis* (KY990015) and a QC of 99%. The cox1 sequence (656bp, GenBank accession no: PQ219693) showed 92.33% homology with *Dirofilaria immitis* (OR434081), with a QC of 92%, obtained from an ocelot (*Leopardus pardalis*) in Brazil. No sequences of *D. tenuis* were available for comparison. Phylogenetic analysis of the 18S, 28 rRNA region, and cox1 gene fragments confirmed assignment to the genus *Dirofilaria* and placed the sequences obtained in the current study within clades containing other *Dirofilaria* spp. sequences available in the GenBank. The Maximum likelihood tree using 18S rRNA sequences, nested Yucatan raccoon filarioid worms within a clade that shared a branch with *D. (N.) striata* (bootstrap 73%). This group also included *D. (N.) repens* and *D. immitis* clades and was clearly distinct from other parasites known to infect other mammals (Fig. 4). The minimum evolution tree generated with 28S sequence showed a support value of 62% for placing our specimens together with *D. immitis* (KY990015), and *D. repens* (KP760376) (Fig. 5). The phylogenetic analysis based on the cox1 sequences placed the *D. (N.) tenuis* sequence in a cluster with *D. immitis* (OR434081, KF692100 and EU159111) and *D. striata* with 95% branch support (Fig. 6).

### 3.5 Parasite prevalence

A total of 162 nematodes were collected from all five examined raccoons, resulting in a prevalence of 100%. The mean intensity was 32.4 (SD: ±12.25), ranging from 18 to 45 parasites per raccoon. Nematodes were mainly coiled in the subcutaneous connective tissues in the ventral region, limbs, and head. However, they were also found to be extended between the muscle fibres of the legs and hips. In four out of five (80%) raccoons, a single female adult worm was found in the heart's right ventricle.

## 4 Discussion

The Americas is an important region for the distribution of *Dirofilaria* species, with several infections reported across different geographic areas from wild and domestic mammals (Dantas-Torres and Otranto, 2013). However, current knowledge remains limited regarding the number of *Dirofilaria* species in tropical geographic regions, such as Southern Mexico, which harbour a large variety of wild carnivores and possess high zoonotic potential (Otranto and Deplazes, 2019).

We have identified our specimens as *Dirofilaria (Nochtiella) tenuis* Chandler (1942), based on morphological characteristics previously defined as distinctive for adult male and female worms of this species (Chandler, 1942; Eberhard, 1978; Orihel and Beaver, 1965). Such characteristics include the presence, distribution size, and shape of longitudinal cuticular ridges (LCR), as well as the arrangement of the caudal papillae in males and the structure of copulatory spicules. Nematodes belonging to the *Nochtiella* subgenus typically exhibit smaller dimensions than *Dirofilaria* species. For instance, females of *Dirofilaria immitis* can reach lengths up to 30 cm. In contrast, females of *D*. (*Nochtiella*) *tenuis* generally reach lengths around 10 cm (Gustinelli et al., 2012), consistent with our specimens. Additionally, *Nochtiella* species have a cuticle with fine transversal striations and prominent longitudinal cuticular ridges throughout the body (Orihel and Beaver, 1965; Wong and Brummer, 1978). Our histologic analysis of transverse sections and SEM showed that our specimens possessed a series of low and smooth rounded ridges arranged in a wavy, broken, and branched pattern. These features are consistent with previous descriptions by Chandler (1942), Orihel and Beaver (1965), and Wong and Brummer (1978). These morphological characteristics are considered of key relevance for distinguishing *D. tenuis* from other *Dirofilaria* and *Nochtiella* species (Gutierrez, 1984), such as *D. ursis*, presenting tall, sharply crested, narrow ridges, and *D. immitis*, having smooth cuticle, with ridges present only in the posterior part of the male (Gustinelli et al., 2012).

Chandler's original description of *Dirofilaria tenuis* (Chandler, 1942) was based solely on female specimens. Orihel and Beaver (1965) described that the caudal papillae in males are paired, numbering up to 15 in total, including 6 to 9 preanal, 4 or 5 postanal, and one pair on the midventral line immediately behind the anus. In our specimens, we observed between 4 and 7 papillae on each side, with the first preanal papillae not always paired, in addition to a lenticular-shaped papilla on the midline adjacent to the upper lip of the cloaca. This lenticular-shaped papilla has also been reported in *D. immitis* (Wong and Brummer, 1978), *D. cancrivori* (Eberhard, 1978), and *D. magnilarvatum* (Price, 1959).

Regarding the copulatory structures of our specimens, both spicules exhibited a high degree of similarity in shape and proportions to those reported by Orihel and Beaver (1965). Their lengths fell within the range described by McIntosh (1954) (Perles et al., 2024; Orihel and Beaver, 1965), with left spicules ranging from 210 to 270 and right spicules from 100 to 130. The microfilariae were approximately 5% shorter and 8% thinner than those reported by Orihel and Beaver (1965) and Sauerman and Nayar (1985) for *D*. (*N*.) *tenuis*, likely due to fixation and mounting techniques.

The SEM analyses of the apical view showed that the number and the arrangement of cephalic papillae and amphids were consistent with the previous descriptions for *Dirofilaria* and *Nochtiella* species made by Wong and Brummer (1978). Nevertheless, the distinctive “face” ornamentations of the *Nochtiella* species, as described by Wong and Brummer (1978), were only exhibited by female specimens. The presence of three different facial ridge patterns in our male specimens suggested the existence of several morphs among males, probably due to intraspecific variation. We consider it unlikely that the observed differences in the facial ornamentations among males and between males and females were due to artifacts derived from specimen processing as they underwent the same fixation, dehydration, and Au/Pd coating process for Scanning Electron Microscopy. In terms of future research, examining males of other species within the *Nochtiella* subgenus will be helpful towards a more accurate characterization of these morphological variations and to address some of the taxonomic issues regarding the accurate number of species in *Dirofilaria*.

Sequence comparison to other *Dirofilaria* species records in the GenBank database showed that our 18S isolate has a higher nucleotide identity to *D. striata* than our 28S and cox1 sequences to *D. immitis*. This identity is probably due to the absence of sequences of *D. (N*.*) tenuis* in GenBank for comparison when this study was conducted. However, morphological and histological observations supported us in fully identifying our material as *D. (N.) tenuis*. Thus, our 18S rRNA, 28S rRNA and cox1 sequences for *D. (N.) tenuis* can be considered as the first genetic material of this species in GenBank. The phylogenetic analyses, which involved other members of the Onchocercidae family, placed the 18S sequence obtained in the current study together with *D. striata,* Molin, 1858, within the *Nochtiella* subtree. This grouping may have occurred because both species belong to the same subgenus. However, *D. striata* adult worms are parasites of wild felines, easily differentiated from *D. tenuis* due to their notably larger size, measuring 28–36 cm for females and 8–10 cm for males.

On the other hand, the 28S and cox1 sequences showed similarity to that of *D. immitis*. This species is distinguished by its affinity for the cardiovascular system of its natural host and notably larger size (Gutierrez, 1984). However, reduced LCR at the midbody of adult *D. immitis* worms (Uni and Takada, 1986) confirms the close taxonomic relationship between species of *Dirofilaria* and *Nochtiella* (Michalski et al., 2010).

Our findings indicate a significantly higher prevalence of *D. (N.) tenuis* in raccoons in this study area compared to previous Knott's test-based studies conducted on raccoons in endemic areas of the Southern United States which reported prevalences to be about 50% (Sauerman and Nayar, 1985; Isaza and Courtney, 1988; Telford and Forrester, 1991). A likely explanation for our study's high prevalence and abundance values can be attributed to the high diversity and abundance of Culicidae mosquito species, which could act as vectors (Garcia-Rejon et al., 2023). Also, suitable animal reservoir populations and adequate climatic conditions may facilitate the transmission of tropical zoonotic diseases in the study area (Haro et al., 2021). However, further studies examining a more significant number of hosts are necessary to accurately determine the prevalence of *D. (N*.*) tenuis* in this region. We suggest that the parasite could be distributed along the Gulf of Mexico coastline. Still, it is clear that this nematode has a remarkably high prevalence in raccoons of the northwestern portion of the Yucatan Peninsula. A more extensive study may better determine its potential distribution and prevalence along the southwest and southern Gulf coast. Since a presumed human case of subcutaneous dirofilariasis due to *D. (N.) tenuis* was reported in Costa Rica (Olivo et al., 2019), it is likely that the geographic range of this species could be broader than previously known.

The prevalence and zoonotic nature of *D. (N.) tenuis* has been well-known in the Southeastern United States for a long time (Sauerman and Nayar, 1985; Vincent et al., 2013). In this region, various aspects of the parasitism of this species have been extensively studied, including its morphology and location in the host's body. Unlike previous reports, in the present study, adult females have been found in muscle fibres and the hearts of most of the examined hosts (80%). The potential health effects on definitive hosts resulting from adult worms living in tissues other than the subcutaneous are so far unknown. In fact, it is plausible that these worms could induce myositis and muscular lesions similar to those produced by *Trichinella* spp. and several other helminthic muscular infections (El-Beshbishi et al., 2012).

Nationwide studies show that Mexico is an endemic region for *D. immitis* (Labarthe and Guerrero, 2005; Zumaquero et al., 2020), where the prevalence of heartworm infection in dogs appears to have increased in recent years (Bolio-Gonzalez et al., 2007). Nevertheless, Manrique-Saide et al. (2010) found that only 6.2% of salt marsh mosquitoes (*Aedes taeniorhynchus*) tested positive for *D. immitis* through PCR from a sample of mosquitoes infected with microfilariae in Celestun, Mexico. For the remaining 93.8% of the mosquitoes, it was not possible to determine the specific identity of the larvae, even though the pan-filarial primers used by the authors could have detected other species like *D. repens*. Thus, these larvae may have belonged to some other *Dirofilaria* species from wild animals.

## 5 Conclusion

The current study has provided seminal data on dirofilariasis in wild raccoons from Mexico, extending the known geographic distribution of *D. (N.) tenuis* to Mexico. Additionally, our findings offer insights into the ecological, morphological, and molecular aspects of this filarioid, representing the first molecular characterization of this zoonotic parasite. Our molecular analyses confirmed the species' placement within the *Dirofilaria* closely related to *D. striata* and *D. immitis*. We found a 100% prevalence of *D. (N.) tenuis* in our sample (5 raccoons), suggesting a possible cardiovascular affinity in 80% of the cases (4 out 5 racoons). This study emphasizes the need for ongoing surveillance and molecular studies to monitor zoonotic diseases.

## CRediT authorship contribution statement

**Aarón Hernández-Núñez:** Writing – review & editing, Writing – original draft, Methodology, Formal analysis, Data curation, Conceptualization. **Víctor M. Vidal-Martínez:** Writing – review & editing, Validation, Resources, Funding acquisition, Formal analysis. **M. Leopoldina Aguirre-Macedo:** Writing – review & editing, Validation, Supervision, Resources, Funding acquisition.

## Declaration of competing interest

The authors declare that they have no competing interests.

## Figures and Tables

**Figure:**
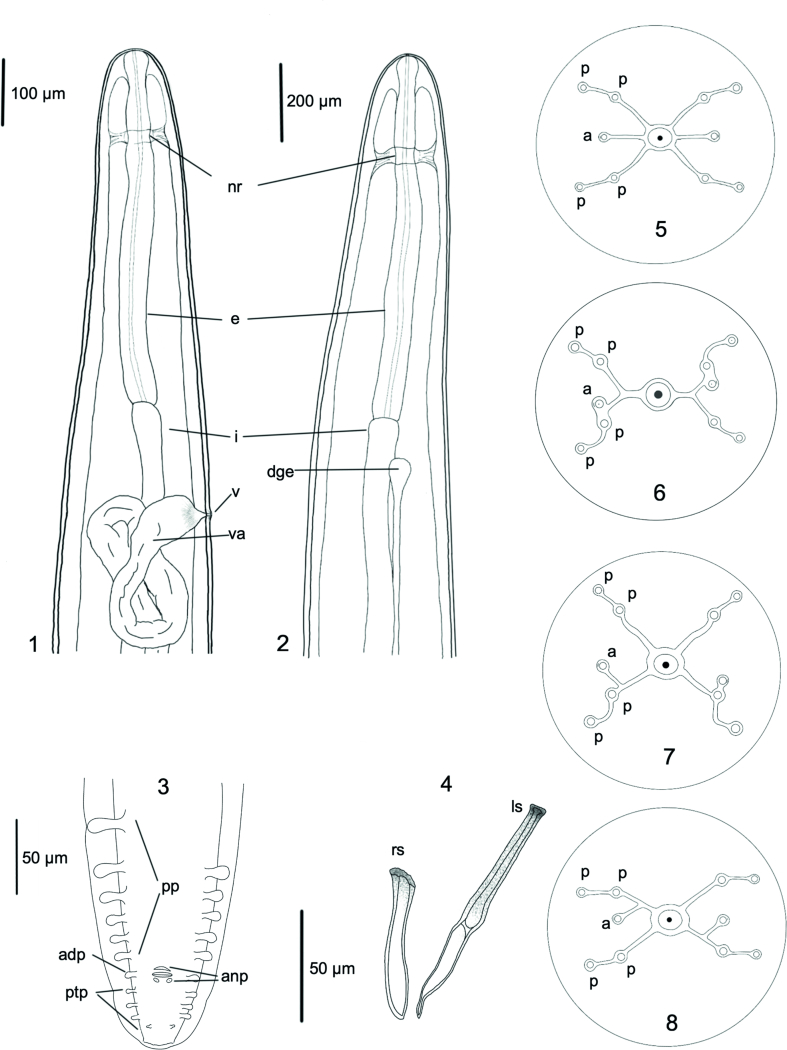
S1. ***Dirofilaria* (*Nochtiella*) *tenuis* overlay tracing and freehand drawings**. Anterior end of female and male nematodes (1 and 2), Ventral view of posterior end of male (3), Ventral view of spicules (4), *En face* view of female (5), *En face* view of males (6,7, and 8). Abbreviations: a, amphidia; adp, adanal papillae; anp, anal papillae; dge, distal genital end; e, esophagus; i, intestine; p, papilla, pp, preanal papillae; ptp, postanal papillae; nr, nerve ring; v, vulva; va, vagina.
